# A Senescence Associated Secretory Phenotype (SASP) in Indolent Systemic Mastocytosis Compared to Healthy Controls

**DOI:** 10.1093/geroni/igaf122.3519

**Published:** 2025-12-31

**Authors:** Jennifer Nicoloro-Santabarbara, Rohan Ravindran, Matthew Giannetti, Dan Dwyer, Michael Lam, Pui Lee, Marianna Castells, Judith Carroll

**Affiliations:** Brigham and Women’s Hospital, Boston, Massachusetts, United States; Brigham and Women’s Hospital, Boston, Massachusetts, United States; Brigham and Women’s Hospital, Boston, Massachusetts, United States; Brigham and Women’s Hospital, Boston, Massachusetts, United States; Boston Children’s Hospital, Boston, Massachusetts, United States; Boston Children’s Hospital, Boston, Massachusetts, United States; Brigham and Women’s Hospital, Boston, Massachusetts, United States; University of California, Los Angeles, Los Angeles, California, United States

## Abstract

Indolent systemic mastocytosis (ISM) is a disease characterized by an abnormal proliferation and hyperactivity of neoplastic mast cells in bone marrow and various organ systems, marked by high rates of cognitive dysfunction, musculoskeletal deterioration, and chronic fatigue; clinical manifestations commonly associated with aging. ISM’s dual identity as clonal and inflammatory may uniquely accelerate biological aging progression with translational relevance to geroscience. However, no studies have explored cellular senescence and related senescence-associated secretory phenotype (SASP) in ISM. We enrolled 40 adults with ISM and 40 age-and sex-matched healthy controls (HC). Plasma protein levels quantified using the Olink 48 inflammatory panel were used to create a 20-protein SASP index. Welch’s t-test revealed ISM patients had 2- to 3-fold greater SASP index scores (M = 3611.6) compared to HCs (M = 1,355.2; t(39.56)= -7.27, p < 0.001). Within the ISM sample, IL1α, a SASP protein, was positively associated with general fatigue subscale of the Multisystem Fatigue Inventory-Short Form (p < 0.05). Patients with CD25+ mast cells, a sign of active disease state, had significantly higher SASP values (M = 3884) compared to patients with inactive disease (M = 2599; t(30.99)=-3.1461, p < 0.05). Findings demonstrate a highly active SASP profile among ISM patients compared to controls, and this pro-SASP profile is more pronounced in patients with active ISM disease. These results point to ISM as a driver of accelerated ‘aging’, informing future clinical and geroscience approaches in this patient population.

